# Insights into the adaptive response of *Arabidopsis thaliana* to prolonged thermal stress by ribosomal profiling and RNA-Seq

**DOI:** 10.1186/s12870-016-0915-0

**Published:** 2016-10-10

**Authors:** Radoslaw Lukoszek, Peter Feist, Zoya Ignatova

**Affiliations:** 1 Biochemistry, Institute of Biochemistry and Biology, University of Potsdam, Potsdam, Germany; 2 Biochemistry and Molecular Biology, Department of Chemistry, University of Hamburg, Hamburg, Germany; 3 Present Address: Division of Plant Sciences/Centre for Gene Regulation and Expression, School of Life Sciences, University of Dundee, Dow Street, Dundee, DD1 5EH UK

**Keywords:** Translation, Ribosome profiling, Transcription, RNA-Seq, Secondary structure, G-quadruplexes, Heat stress response

## Abstract

**Background:**

Environmental stress puts organisms at risk and requires specific stress-tailored responses to maximize survival. Long-term exposure to stress necessitates a global reprogramming of the cellular activities at different levels of gene expression.

**Results:**

Here, we use ribosome profiling and RNA sequencing to globally profile the adaptive response of *Arabidopsis thaliana* to prolonged heat stress. To adapt to long heat exposure, the expression of many genes is modulated in a coordinated manner at a transcriptional and translational level. However, a significant group of genes opposes this trend and shows mainly translational regulation. Different secondary structure elements are likely candidates to play a role in regulating translation of those genes.

**Conclusions:**

Our data also uncover on how the subunit stoichiometry of multimeric protein complexes in plastids is maintained upon heat exposure.

**Electronic supplementary material:**

The online version of this article (doi:10.1186/s12870-016-0915-0) contains supplementary material, which is available to authorized users.

## Background

Environmental stress or suboptimal growth conditions reduce cell viability and require an immediate but specific response in order to maximize the survival of the whole organism. Particularly, plants are constantly exposed to changing environmental conditions and are under threat of severe adverse conditions. On the subcellular level, heat exposure changes membrane fluidity [1, 2] and protein stability [3, 4] which consequently alter photosynthesis [5] and central metabolic activities [6]. Plants are highly sensitive to temperature stress and respond over different time scales [7–10]. One of the most potent steps to regulate heat stress response has been suggested to occur at the level of transcription [11]. Long heat exposure triggers epigenetic changes, some of which are conserved between yeast and plants indicating that these stress response mechanisms are evolutionarily conserved among organisms [8]. Ultimately, proteins mediate stress response and their levels have to be rapidly adjusted to ensure cell adaptability and survival particularly under prolonged stress.

Gene expression is subject to extensive regulation, including transcription, mRNA degradation, translation and protein degradation, each of which operates on a different temporal regime [12–14]. Translation is a downstream process of transcription and provides the opportunity to rapidly adjust protein concentration in response to external stimuli [15]. Although transcriptional reprogramming upon heat exposure has been addressed in plants, little is known for the role of translation. Does translation complement transcription in shaping the heat stress response?

Advances in massively parallel sequencing platforms and approaches to capture ribosomal position with nucleotide resolution, i.e. ribosome profiling [16], precisely capture gene expression at the level of translation. Combined with RNA-Seq to measure changes in mRNA population [17], the transcriptional and translational responses can be deconvoluted. Ribosome profiling has been successfully applied in mammalian systems, for example to study the effect of heat [18], oxidative [19] and proteotoxic stress [20] on translation in mammalian systems. The depth of those approaches revealed unprecedented aspects in the stress response programs which were not detected with a single sequencing method. The applicability of ribosome profiling technology in plants has been recently demonstrated by two studies assessing the global expression reprogramming in *A. thaliana* during dark to light transition [21] and the response to hypoxia [22]. We used the combined approach of ribosome profiling (Ribo-Seq) and RNA-Seq to assess the response of *A. thaliana* to prolonged heat stress. Our study reveals a complex picture of adaptive response in plants and provides a rich resource for future hypothesis testing.

## Results

### A subset of genes shapes the plant adaptation to thermal stress

**Fig. 1:**
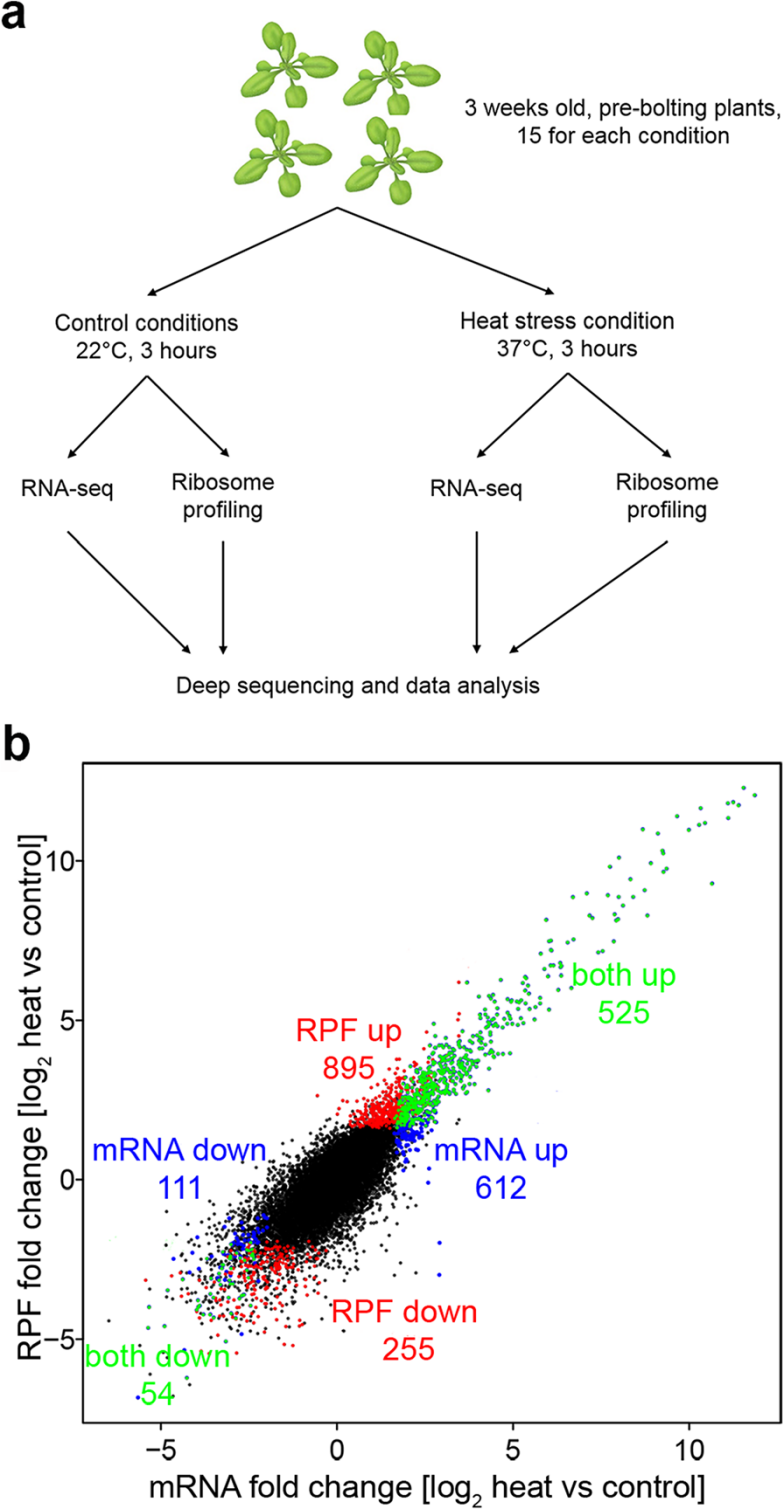
The expression of a sizeable fraction of genes changes at either transcriptional or translational level. **a** Scheme of the experimental set-up. Each set (control and heat stress-treated plants) comprises 15 plants. **b** Differential expression analysis using DESeq with FDR of 0.1. Genes with changes in mRNA expression only are designated in *blue*, those with RPF changes only (mRNA reads unchanged) in *red* and genes with simultaneous changes in both, mRNA and RPF, are highlighted in *green*. The number of genes up- (*up*) or down-regulated (*down*) in each group upon exposure to heat are included. GO analysis (DAVID) of these gene groups is summarized in Additional file 4

To monitor the adaptive reaction we exposed wild-type *A. thaliana* plants (Columbia-0) to a prolonged heat of 3 h at 37 °C. To provide a high-resolution view of the cellular programs that counteracts thermal stress at both translational and transcriptional level, we isolated ribosome-protected fragments (RPF) and total RNA from leaves, and subjected both to deep sequencing. The sequencing of the RPFs (Ribo-Seq) is informative on the translational activities of the cell [16, 23] while the total RNA sequencing (RNA-Seq) [17] reports on transcriptional activities. These were compared to untreated plants growing at permissive ambient temperature (Fig. 1a). RPFs were generated by nuclease digestion of polysomes into monosomes with high reproducibility between biological replicates (Additional file 1a, b). The unambiguously mapped mRNA and RPF reads were normalized by the total number of mapped reads (rpm) or reads per kilobase per million of the total mapped reads (rpkm). We spiked each RNA-Seq experiment with external RNA-standards (Ambion) whose sequence did not align anywhere in the plant genome; the spike-ins were used to determine the detection limit (i.e. the minimal rpkm) in each experiment. In both biological replicates the detection limit in RNA-Seq and Ribo-Seq was 2 rpkm. In general, the RPF density correlated well with the mRNA reads density (Additional file 1c) suggesting a coordination of transcriptional and translational programs at control temperature growth. Following heat stress, RPF and mRNA reads were still well-correlated overall, albeit slightly reduced compared to the control growth conditions, and suggested that a significant translation activity was presented by the heat-exposed plants (Additional file 1d). The polysome fraction, which comprises actively translating ribosomes, is similar to that of the control plants and only marginally reduced in the fractions of heavy (>5) polysomes (Additional file 1e, f). Only a small increase of the monosome peak was detected (Additional file 1e); an increase of the monosome peak is usually observed under acute stress [24]. Note that we could not resolve the single ribosomal subunits (40S and 60S); 60S appeared as a shoulder of the 80S or monosome, and thus we could not estimate the ribosomal drop-off (Additional file 1e). Also, the total RNA used in the polysomal profiles varied as it was normalized by the mass of the used plant material and thus reflects the different RNA content of the plants grown at various ambient temperature.

Overall, for the majority of genes that are translationally active under heat (i.e., for which RPFs were detected), we found a positive linear log-log correlation with changes in their mRNA reads (Additional file 1d) suggesting that the adaptive response is shaped in a coordinated manner between transcription and translation.

However, a sizeable fraction of genes differed in their expression (i.e. exhibited disproportionate ratios of the mRNA to RPF reads) (Additional file 2a, b). Those gene groups may provide candidates whose expression is controlled either transcriptionally or translationally. Hence, we used differential expression analysis (DESeq) to compare the mRNA and RPF counts of each gene expressed in control plants grown under ambient temperature to that in the plants exposed for 3 h to 37 °C (Fig. 1b). The confidence intervals for the fold-change analysis were set based on the reproducibility of the biological replicates for the control plants (Additional file 1a, b). DESeq analysis considers as expression level the sum of all RNA or RPF reads over a transcript but is insensitive to the distribution of reads along a transcript. If translation of a gene is enhanced, we expect increased RPF reads along the entire open-reading frame (ORF) length. We reasoned that if a gene is uniformly translated with no detectable heat-induced stalling over certain position(s) within the CDS, the counts of the RPF reads between the two halves of a gene should be equal. Notably, RPFs were nearly symmetrically distributed between the two halves of a coding sequence (CDS) of genes expressed under heat exposure and resembled the uniform distribution between the two halves of the mRNA (Additional file 2c, d), suggesting that higher total RPF reads truly report on enhanced expression of those genes under heat stress.

The DESeq analysis revealed co-directional changes in the mRNA and RPF counts for 579 out of 14,246 genes (525 upregulated and 54 downregulated; green designated, Fig. 1b). A sizeable fraction of genes showed only changes in the mRNA (723 genes, blue designated, Fig. 1b) or in RPF (1150 genes, red designated, Fig. 1b). For each of the groups with altered RNA expression or translatability (i.e., altered RPF reads), we performed enrichment analysis using DAVID (Additional file 3). The most prominent groups among those upregulated at transcriptional and/or translational level (i.e. significantly higher mRNA and/or RPF read counts) were genes involved in the heat stress response and protein folding (e.g. chaperones and heat-shock proteins). Interestingly, although the plants were exposed to heat for 3 h, which should elicit the adaptive response to heat stress, the mRNA of key heat shock proteins was very high (Additional file 4a, b). In contrast, groups comprising genes related to the chromatin structure, cytoskeleton organization, cell wall synthesis, cell cycle, and anabolic process were mostly down-regulated at transcriptional and/or translational levels (Additional file 3). Together, prolonged exposure to heat stress resulted in large changes in gene expression and reprogramming of both transcriptional and translational activities of the plants that are likely to shape their survival under sub-optimal growth conditions.

### Genes with lower secondary structure propensity in 5′ start vicinity are translated under thermal stress

**Fig. 2:**
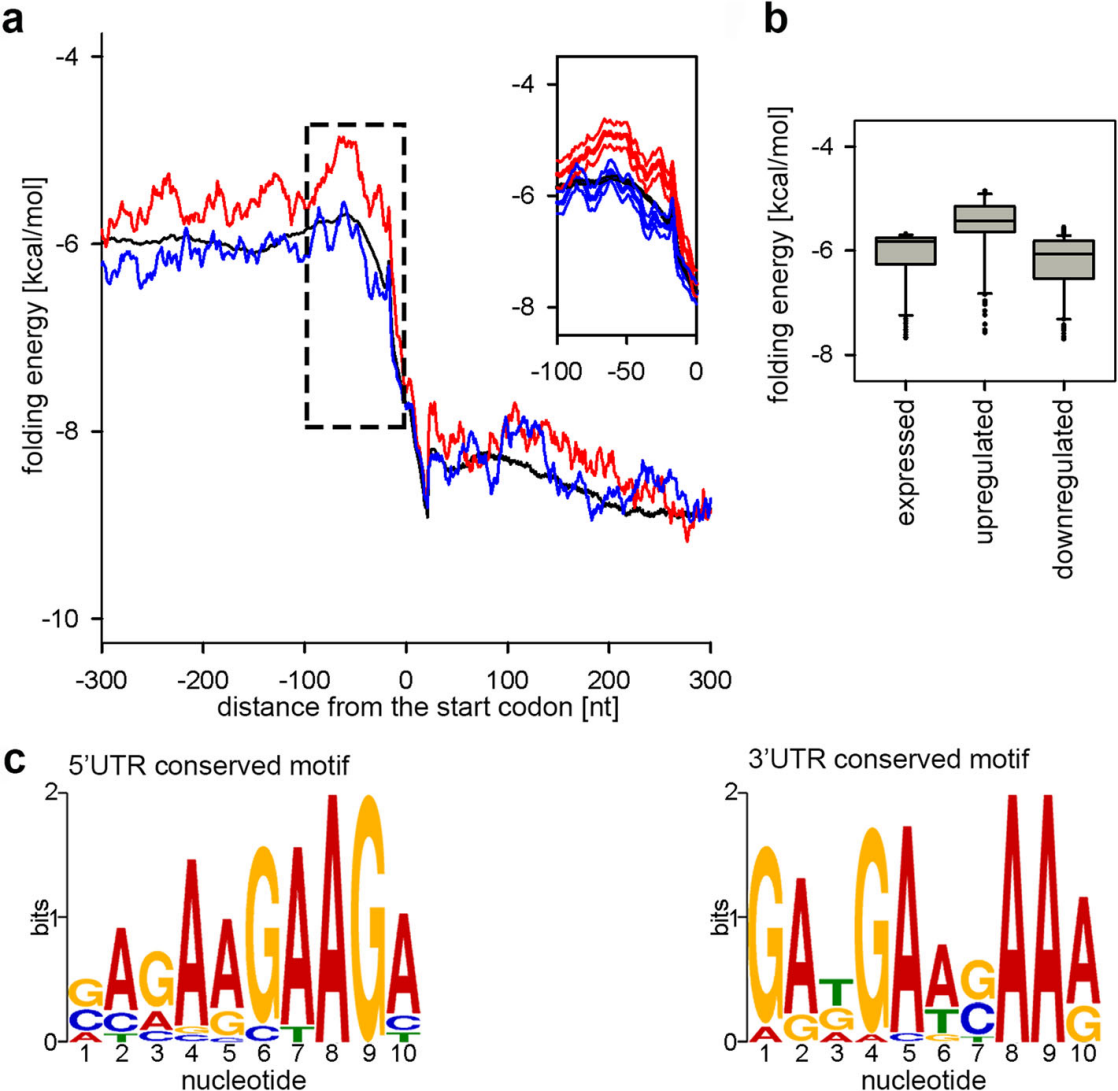
Genes translationally upregulated under heat stress have much lower propensity to form secondary structure in the vicinity of the start codon. **a** Average folding energy of translationally upregulated (*red line*) and downregulated (*blue line*) genes under heat stress compared to all expressed protein-coding genes. Only in the marked area (inset) the *curves* shows significant difference (*p* = 2.2*10^−16^ (median averaged from a two-sample Mann-Whitney test)). The *thin lines* in the same color denote the standard deviation for each position. Position 0 is the first nucleotide of the start codon of each genes. **b** Box plot of the distribution of the folding energy of the genes translationally up- or down-regulated under heat stress compared to all expressed genes. The region −100 nt upstream of the start till the start codon (position 0) is considered. **c** Sequence motif analysis of 5′UTRs (*left logo*) and 3′ UTRs (*right logo*) of genes translationally upregulated under heat stress

Next, we addressed whether the gene set that is preferentially translated under heat stress (red marked gene groups, Fig. 1b) bears some common secondary structure features to facilitate their translation. We calculated the folding energy in the mRNA sequences flanking the translation initiation start of two groups of genes, e.g. with increased (translationally upregulated) and decreased (translationally downregulated) ribosome density. Typically, the folding profiles of all mRNAs (Fig. 2a, black line) exhibited reduced folding stability and fewer paired nucleotides in the 5′ UTRs compared to the coding sequence (observed as a lower folding energy in the profile). For translationally upregulated genes under heat, the folding energy upstream of the start codon (up to 100 basepairs (bp)) was significantly higher (Fig. 2a, red line) than that of the remaining genes in the genome (Fig. 2a, black line) and that of the translationally downregulated ones (Fig. 2a, blue line). Further downstream of the start codon, along the CDS, the folding energy relaxes to the mean folding profile of all genes (Fig. 2a). The folding energy profile of genes translationally downregulated under heat did not differ from that of the remaining genes in the genome (Fig. 2a, b). This implies that the response to heat stress in plants at translational level is shaped, at least in part, by a selection of a subset of genes with lower propensity to form secondary structure upstream of the translation start. Our attempt to verify the predicted folding patterns with experimentally derived RNA secondary structure data [25, 26] was not successful. Both studies [25, 26] were conducted under normal growth conditions in which the heat shock responsive genes show very low expression level, hence the read coverage was insufficient to obtain reliable secondary structure scores for those genes.

To address the question as to whether RNA-binding proteins contribute to translational regulation through binding the 5′ and 3′ UTRs, we performed a motif search in the UTR sequences of the genes which were translationally upregulated (i.e. changed RPF reads, but unchanged mRNA reads) upon the heat stress. In total, 55 genes (out of 895 heat upregulated) exhibited an increased number of RPF in their 5′UTR and in 23 of them we detected a conserved A/G-rich motif (Fig. 2c). Similarly, in 82 genes the RPFs in their 3′ UTRs increased upon stress and in 23 of them we also detected the A/G-rich motif (Fig. 2d).

### G2-quadruplexes in the UTRs may also control gene expression under stress

**Fig. 3:**
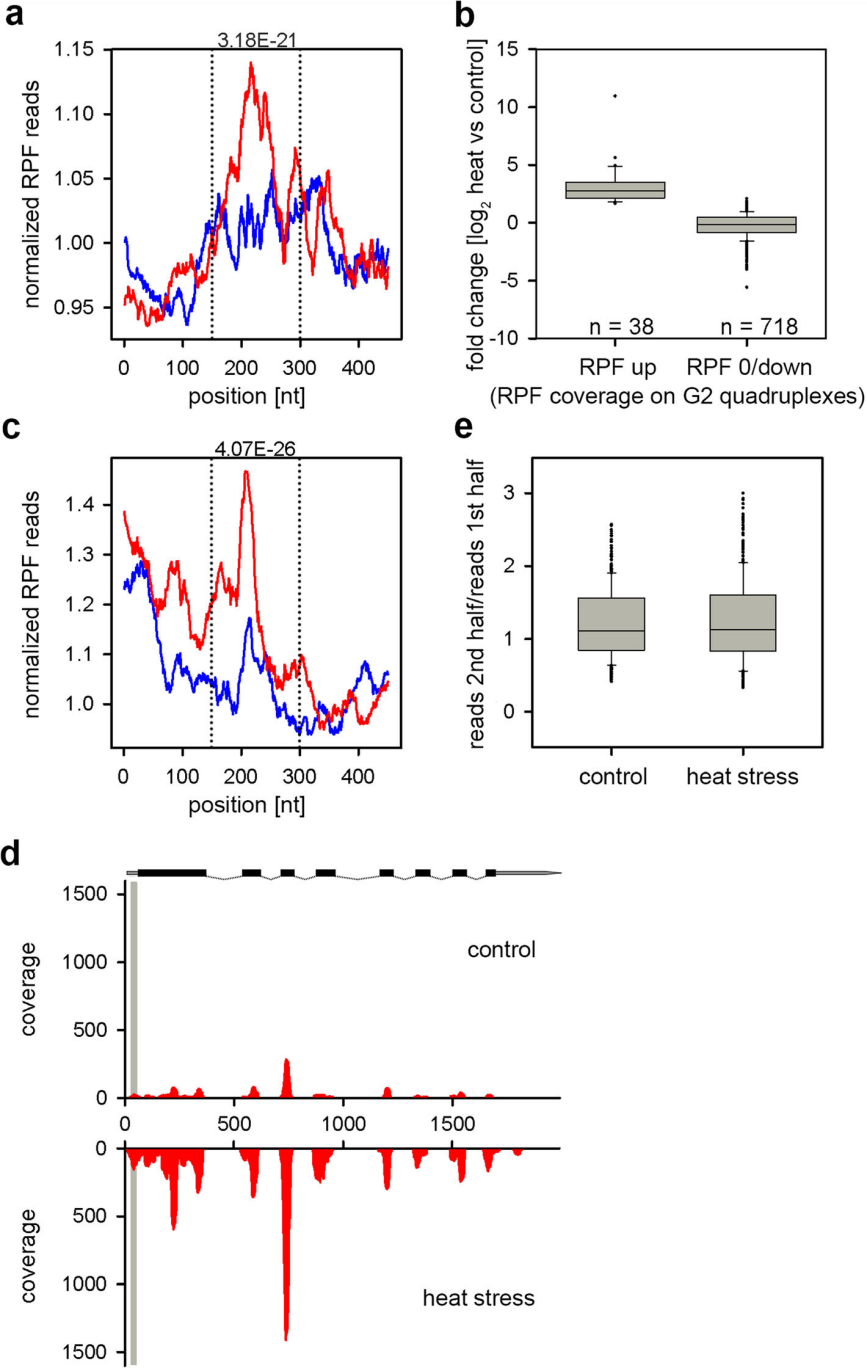
The presence of G2 quadruplexes in the UTRs correlates with the expression of genes under heat stress. **a**, **c** Higher cumulative values of the normalized RPF reads to the mRNA reads at each position under heat stress-exposed plants (*red*) compared to the control plants (*blue*) over the positions of the putative G2 quadruplexes in the annotated 5′ UTRs (**a**) and 3′ UTRs (**c**). RPF coverage (rpm) was normalized to the mRNA reads at each position and each gene in the set is equally weighted. The first nucleotide of the G2 quadruplexes is at position 200. *p*-values (*on the top of the plots*) were calculated with the Wilcoxon signed rank test. **b** RPF fold change analysis of genes with G2 quadruplexes in their 5′ UTRs. Genes upregulated under thermal exposure (RPF up) were compared to genes with G2 quadruplexes but unchanged or downregulated under stress (RPF 0/down). Only reads in the CDSs were considered in this analysis. *n* denotes the gene number in each group. **d** RPF coverage profile of gene At3g56090 under heat stress exposure compared to its profile in the control plants. The position of the G2 quadruplex in the 5′UTR is shadowed in *gray*. In the gene scheme over the profiles: *black*, CDS; *gray*, 5′ or 3′ UTRs; *gray dashed line*, introns. **e** Comparison of the reads in the first vs. second halves of genes with G2 quadruplexes in their 3′ UTRs

We next analyzed each gene translationally upregulated under heat stress (red and green marked gene groups, Fig. 1b) for putative G-quadruplex structures in the CDS, 5′UTR or 3′ UTR. This analysis was motivated by a bioinformatic study which has identified more than 1200 quadruplexes with a G_3_-repeat sequence motif and ∼ 43,000 with a G_2_-repeat sequence motif in plant transcriptomes with yet unknown function [27]. The following sequences were considered in our search for G2 - G_2_N_1-7_G_2_N_1-7_G_2_N_1-7_G_2_, for G3 - G_3_N_1-7_G_3_N_1-7_G_3_N_1-7_G_3_ and for G4 - G_4_N_1-7_G_4_N_1-7_G_4_N_1-7_G_4_. We found no G4 quadruplexes and only a few G3 quadruplexes in the 5′ and 3′ UTRs. However, we identified many G3 quadruplexes in the CDS (515 in total) and G2 in the 5′UTR (975), CDS (17,845) and 3′UTR (1479), respectively. We reasoned that if a G-quadruplex plays a role in heat response and controls expression of distinct mRNAs upon stress, we would observe different translation (i.e. differences in the RPF coverage) in the vicinity of a quadruplex structure between plants exposed to heat compared to the control plants. We compared the read coverage 200-bp upstream and 250-bp downstream of the first base of each quadruplex. While we observed no difference in the RPF coverage around quadruplexes in the CDS (Additional file 5), the RPF coverage around G2 quadruplexes in both 5′ and 3′ UTRs was clearly higher in the heat stress group (Fig. 3a, c). Intriguingly, in the genes upregulated under heat stress (both up, green, and RPF up, red, in Fig. 1b) the higher RPF coverage along the predicted G2 quadruplexes in the 5′UTR correlated with their higher expression under heat than in the control plants (Fig. 3a, b, d). This suggests that a direct relationship might exist between the G2-quadruplexes and translatability of the downstream CDS under heat stress.

The effect of the G2 quadruplexes in the 3′UTRs of genes upregulated under heat exposure is unclear; they did not contribute to the stability of the mRNA under heat stress, i.e. the mRNA reads for the two halves of a gene remained unchanged under stress (Fig. 3e).

### Detection of an alternative transcript and ORF upon stress exposure

**Fig. 4:**
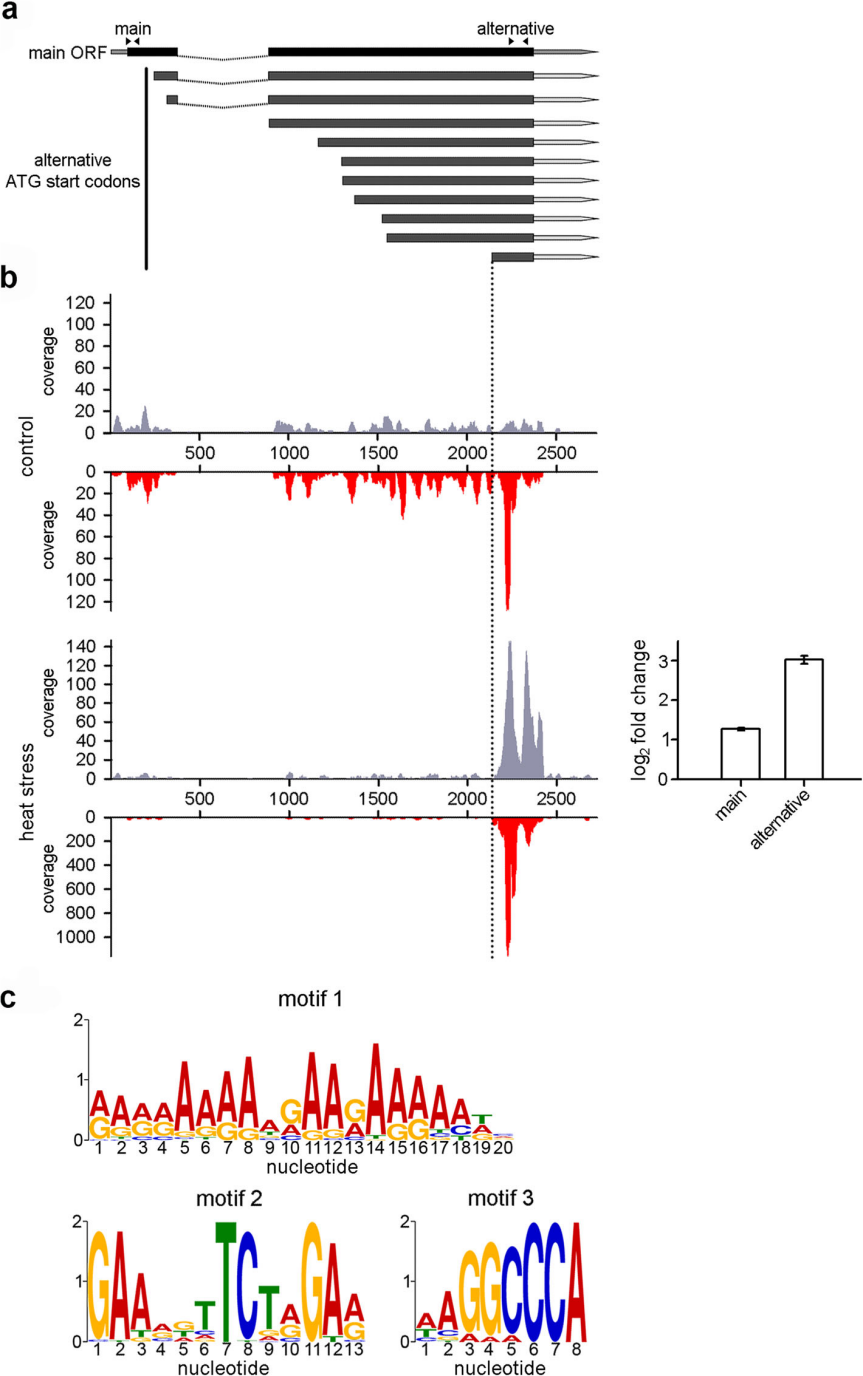
Alternative transcript from the At1g76880 gene is highly expressed under heat stress. **a** Schematic of the putative ORFs of the At1g76880 gene. *Black*, CDS; *gray*, 5′ or 3′ UTRs; *gray dashed line*, intron, and *arrow heads* above the gene model: locations of the qRT-PCR primers. **b** In addition to the main transcript, a transcript encoding 88-amino acids long peptide is detected in both ribosome profiling and RNA-Seq data sets (*plots on the left*) as highly expressed at both transcriptional (mRNA reads, *gray*) and translational level (RPF reads, *red*) under heat stress. The *dashed vertical line* denotes the start of this additional ORF. Note the different scale of the coverage profile in the heat vs. control condition. qRT-PCR verification of the expression of the alternative transcript under stress (*right panel*) using primer pairs spanning the main and alternative transcript designated in panel a (*arrow heads*). **c** Three distinct recurring motifs are present within 1 kb-region upstream of the initiation of the alternative ORF (upstream 2100 nt) and multiple heat shock responsive genes as revealed by MEME motif analysis

In our analysis of the RPF distributions between the first and a second half of a gene we noticed an outlier, At1g76880, with largely asymmetric distribution of the reads in the second half upon exposure to heat. A closer look in the read distributions revealed that in the control plants, At1g76880, which encodes a double-helix repeat protein, showed a relatively uniform mRNA coverage over the main CDS, while RPFs accumulated starting at nucleotide (nt) position 2195 (Fig. 4a, b). Upon induction of heat stress, the expression of this short 3′-terminal fragment starting at 2195 nt increased at both transcriptional (i.e. increased mRNA reads) and translational level (i.e. increased RPF reads). In the vicinity of the 2200 nt we detected an in-frame ATG which may serve as a new translation start (dashed vertical line, Fig. 4b). Although an alternative translation start of the same mRNA transcript may plausibly explain the RPF enhancement, it cannot explain the increase of the mRNA reads. qRT-PCR analysis using primers targeting the main and alternative transcript corroborated the RNA-seq data and indicated specific upregulation of the alternative transcript under heat stress (Fig. 4b). Moreover, only one splicing variant of At1g76880 is annotated in the TAIR 10 data base. Furthermore, additional in-frame AUG codons in the CDS (Fig. 4a) did not correlate with any increase of RPF reads at those corresponding positions (Fig. 4b).

Within the region 1 kb upstream of this alternative ORF and of the heat shock responsive genes we performed a sequence motif search to extract putative sequences that may serve as putative transcription factors binding sites. Interestingly, we identified motifs which share conserved features with motifs found in the promoter regions of the known heat-stress responsive genes (Fig. 4c) which were transcriptionally induced upon heat stress in our data set. The presence of motif 2 bears significant resemblance to the heat shock promoter element AGAAnnTTCT recognized by heat shock factors in *Arabidopsis* [28] supports the idea of this alternative transcript being heat-responsive. This alternative transcript with a start at 2195 nt encodes an 88 amino-acid long peptide/protein with high overrepresentation of positively charged amino acids; proteins with overrepresented charged amino acids may play a protective role under stress, e.g. scavenging reactive oxygen species. Although it remains to be determined whether the expression of this alternative transcript generates a viable protein or peptide, our results underline the potential of Ribo-Seq in determining alternative ORF or proteins resulting from alternative, independent translation initiation which differ from the start of the main transcript.

### Stoichiometry of protein complexes in chloroplasts under heat exposure

**Fig. 5:**
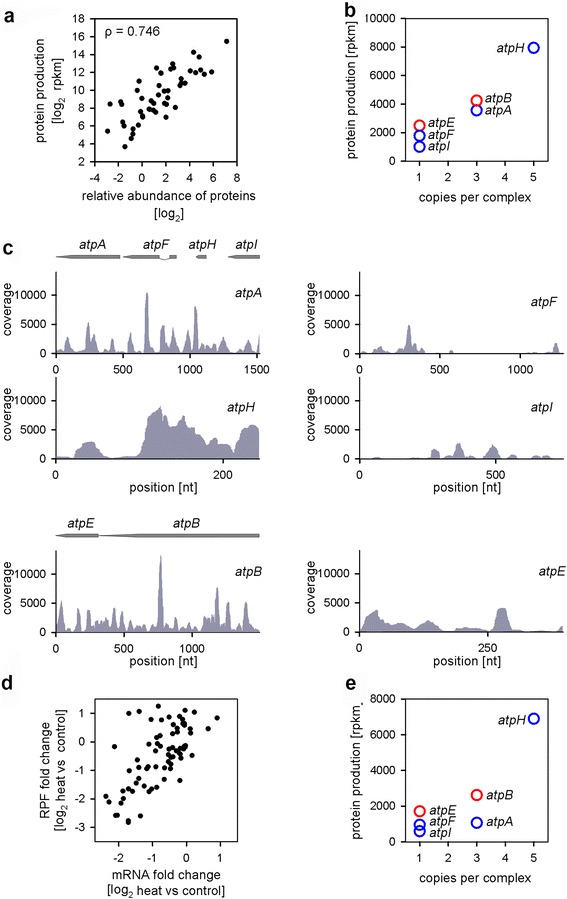
Impact of heat exposure on the expression of protein complexes in chloroplasts. **a** Correlation between protein production (RPF reads) and protein abundance determined by mass spectrometry [31]. Spearman coefficient ρ = 0.746. **b** The protein production of ATP synthetase subunits correlates with the assembled subunit stoichiometry. The genes belonging to each of the two operons are color-coded in *blue* and *red*. **c** Coverage plots for each of the subunits of the ATP synthetase complex under control growth. Note that the y-axes are in uniform scale. Schematic of the gene organization in the two operons is included over the plots. **d** Fold changes of RPF and mRNA of all chloroplast ORFs. Each dot represents a single protein or protein subunit. **e** Heat exposure disproportionately reduces the production of ATP synthetase subunits (compare with panel **b**)

In the ribosome profiling experiment we did not select only for cytoplasmic ribosomes, but extracted the total fraction of the all ribosome-bound mRNAs, including those of the chloroplasts. In each sequencing data set, 35–45 % of the total uniquely mapped RPFs were mapped to the chloroplast genome. As the chloroplast genome is relatively small −117 total genes including 87 protein-coding genes – the coverage in the ribosome profiling is very good. The majority of the plastid-encoded genes encode single subunits in large protein complexes. Some of them are encoded in operons within one polycistronic mRNA in a fashion similar to bacterial operons [29] and are suggested to coordinate the expression of functionally related proteins. However, a large fraction of genes encoding subunits of protein complexes do not reside within the same operon, raising the question as to whether their translation maintains the stoichiometry needed for the protein complexes. Thus, we next analyzed the stoichiometry of protein complexes using the total RPF reads per gene per unit length (rpkm) as defined in [30]. The underlying assumption of this analysis is that each ribosome (or here RPF) is producing a protein and the total protein production is determined as the average ribosome density over the CDS. Although this measure is not perfect as it provides an upper bound for protein levels [30] as it does not consider protein degradation and ribosomal drop-off during synthesis, our measures of protein production using this approach (Fig. 5a) agreed well with published data on protein abundance in chloroplasts [31].

We next used this measure to evaluate the production of stable multiprotein complexes with known stoichiometry (Fig. 5b and Additional file 6a). Remarkably, for the ATP synthetase complex, which has the most complex stoichiometry, the protein production of each subunit quantitatively reflected its stoichiometry within the complex (Fig. 5b). The ribosome density of each ORF was different despite comprising the same polycistronic mRNA (*atpA/E/I/H* and *atpB/F* are the two operons, Fig. 5c). The mRNA levels of these two operons were similar as confirmed by RNA-Seq analysis, further validating that differences in the stoichiometry might be controlled at the level of translation. For some complexes, which are encoded mostly on different polycistronic mRNAs, the ratio of protein production of some subunits differed from their stoichiometry (Additional file 6) and suggests an additional regulation mechanism at the level of degradation.

The expression of protein-coding genes in chloroplasts changed under heat exposure and for the majority of the ORFs changes in mRNA levels were co-directional with changes in transcription (i.e. mRNA reads) and translation (i.e. RPF reads) (Fig. 5d). Strikingly, the production of the subunits within one protein complex changed disproportionately, even for those upregulated under heat stress (Fig. 5e and Additional file 6b) which could suggests that the different susceptibility to degradation of various subunits may additionally change the abundance of the subunits under stress.

## Discussion

Here we analyze the adaptive response of *A. thaliana* to prolonged heat exposure (3 h at 37 °C) at both transcriptional and translational level using RNA-Seq and deep sequencing of RPFs of nuclear- and chloroplast-encoded genes. The plant habitat suggests that a typical heat exposure is long, for example for several hours in a summer midday. The expression changes of the majority of nuclear-encoded genes are modulated in a coordinated manner at the transcriptional and translational levels. While at early time points of heat exposure, i.e. between 15 and 45 min, translation is globally downregulated and stress response is counteracted mainly by transcriptional programs [32–34], our results show that prolonged exposure to stress (3 h) activates translational programs which shape the adaptive response. At prolonged exposure to heat stress the majority of the genes are transcribed and translated in a coordinated fashion, but a sizeable set of genes opposes this trend and instead shows only changes at the level of translation. Among those translationally regulated transcripts we detected several shared features which are likely candidates to regulate their expression. The A/G-rich motifs in the 5′ or 3′ UTRs of the translationally upregulated genes resemble sequences identified as RNA-protein binding motifs [26]. The presence of relatively conserved A/G-rich motifs to which most likely the same RNA-binding protein binds would allow coregulation of the expression of those transcripts [26]. Another common feature among the genes translationally upregulated under heat stress is their lower propensity to form secondary structure, likely to facilitate ribosome binding and enhances translation [35, 36]. Furthermore, some of the transcripts preferentially translated under heat contain a putative G2 quadruplex in their 5′UTR. The increased RPF reads over the quadruplex structures correlate with the enhanced expression in the downstream CDS, suggesting a role in activating translation of the downstream ORF with a yet unclear mechanism. This is in line with an earlier observation that transcripts with highly structured 5′UTRs are enriched upon heat exposure [37]. Although the type of the secondary structure in the 5′UTR is not specified [37], the authors suggest a mechanism to sense heat in a similar fashion to the riboswitches in bacteria [38].

The duration of heat stress has different effects on transcriptional and translational programs. The sequence of response seems to follow a conserved pattern in microorganisms and mammalian systems. An initial reaction upon acute heat stress comprises global translational downregulation [18, 20] and a quick transcriptional activation of heat-shock proteins [39]. This first transcriptional burst is followed by an adaptive response which includes reprogramming of many cellular activities with a prominent activation of the heat-stress response, relating to protein folding and degradation, at both translational and transcriptional levels [39]. Previous studies in *A. thaliana* addressing short term (10–45 min) heat exposure provide evidence that translation is greatly inhibited [33, 34, 40]. By contrast, our data show that under prolonged heat exposure (3 h) translation is fully active (the polysomal fraction is only marginally changed, Additional file 1e), suggesting a common pattern of stress response between *A. thaliana*, mammalian cells and microorganisms. Despite the global translational repression under short term heat stress, some transcripts are selectively translated, which includes genes involved in transcriptional regulation, chromatin structure rearrangements, mRNA degradation, salicylic acid-mediated signaling and protein phosphorylation are activated under short term heat exposure [9, 33, 34, 40]. In contrast, extended heat stress (3 h) activates genes involved in heat-stress response and protein folding and deactivates genes related to the chromatin structure, cytoskeleton organization, cell wall synthesis, cell cycle, and anabolic processes. Strikingly, the most prominent gene groups translated in both short term [34] and extended heat stress (Additional file 3) as also observed for mammalian systems [39] suggesting a common features in maintaining heat stress among organisms.

Translation in chloroplasts shares many features with bacteria, including Shine-Dalgarno-driven initiation and polycistronic mRNAs. The prevalence of genes encoded in polycistronic transcripts in prokaryotes has been suggested as a mechanism to couple translation and control the stoichiometry of the single subunits in multisubunit complexes or to control the level of proteins with related functions in metabolic pathways [29]. Although the premise might be true for some examples, it does not explain how a higher number of subunit copies can be achieved downstream in the operon (the example with ATP synthetase, Fig. 5c). Furthermore, in bacteria translation rates among genes within the same operon are only weakly correlated [41] and the architecture of several metabolic pathways is robust against variations in the single proteins, suggesting that a precise translational coupling may not be crucial for their performance [42, 43]. In plastids of *A. thaliana*, we detected a clearly decoupled translation of single genes within an operon; each single ORF within one polycistronic message is initiated in an independent manner with distinct yield (the example with ATP synthetase, Fig. 5c). Similar observation has been made in plastids of maize [44] and in bacteria [30]. Rather than coupling of their translation, the differential synthesis rates within one message [45, 46] and/or degradation rates of the subunits [30] determine the precision to achieve the balanced stoichiometry of subunits. Under thermal stress, we observed variations in the translation rates of single subunits (Fig. 5e and Additional file 6) which lead to alterations in the production of the single subunits that deviated from the expected stoichiometry. Translation rates may change disproportionately because of temperature-dependent variations in the diffusion properties of the different translation components [47]. Precise control of the stoichiometry of protein complexes at elevated temperatures could also be established by differential degradation of the subunits at elevated growth temperatures [48–51].

## Conclusions

In summary, our data unravel new aspects in the adaptive response of *A. thaliana* to heat stress at the level of translation. The adaptation to heat exposure is fine-tuned by a sizeable set of genes whose translation is most likely regulated by different secondary structure elements. Furthermore, the Ribo-Seq and RNA-Seq data provide a vast resource of the *Arabidopsis* transcriptome and translatome at permissive temperature (i.e. control growth conditions) and under heat stress that can inform future experiments focused on understanding transcriptional and translational regulation of nuclear and plastid-encoded genes.

## Methods

### Plant growth and heat treatment

Wild-type Col-0 plants (The European Arabidopsis stock center NASC, ID N1092) were grown on soil in a greenhouse in a long-day condition (16 h/8 h, lamps Philips Master HPI-T Plus, 400 Watt Philips SON-T Agro, 400 Watt, light intensity ~140 μmol.m^−2^.s^−1^, humidity 60 %). The leaves from 15 pre-bolting, 3-week-old plants (stage 3.50 according to [52]) were exposed for 3 h at 37 °C with constant humidity of 60 % (heat stress; [7]), or at 22 °C and served as control. The choice of the duration of the thermal stress was also driven by the availability of data from a previous study addressing transcriptional changes using microarray technology [7]. Plants were pooled, leaves harvested and immediately frozen in liquid nitrogen and stored for further treatment. The total RNA was extracted using TRIzol reagent (Invitrogen), cDNA was synthesized with random hexamers and RevertAid™ H Minus First-Strand cDNA Synthesis Kit (Fermentas) and analyzed with qRT-PCR using Power SYBR Green Master Mix (Life Technologies) and with the following primer pairs: for HSP70 (At3g12580) 5′-CCGTCTTCGATGCTAAGCGTCT-3′ and 5′-AACCACAATCATAGGCTTCTCACC-3′; HSP101 (At1g74310) 5′-ATGACCCGGTGTATGGTGCTAG-3′ and 5′-CGCCTGCATCTATGTAAACAGTG-3′; HSFA2 (At2g26150) 5′-TCGTCAGCTCAATACTTATGGATTC-3′ and 5′-CACATGACATCCCAGATCCTTGC-3′; UBQ10: 5′-AAAGAGATAACAGGAACGGAAACATAGT-3′ and 5′-GGCCTTGTATAATCCCTGATGAATAAG-3′; At1g76880 main transcript 5′-ACGATGATGCAACTGGGTGGTG-3′ and 5′-AGCAGTTGTGACGGTTGTAGCC-3′; At1g76880 alternative transcript 5′-TCGGAGCAGAACTTTGATGATGA-3′ and 5′-GCTCGAACTCACCTCCTTCCTC-3′.

### Polysome profiling

Polysomes were isolated according to [53] with some modifications. Briefly, 10 g of leaf material was thawed in polysome extraction buffer (0.2 M Tris pH 7.4, 0.2 M KCl, 0.025 M EGTA, 0.035 M MgCl_2_, 1 % Brij-35, 1 % Triton X-100, 1 % Igepal CA 630, 1 % Tween 20, 1 % DOC, 1 % PTE, 5 mM DTT, 1 mM AEBSF, 100 μg/mL cyclohexamide, 100 μg/mL chloramphenicol), homogenized using a glass homogenizer, filtered through four layers of sterile cheese cloth and two layers of sterile Miracloth (Calbiochem) and incubated on ice for 10 min. The supernatant after centrifugation (4 °C, 16,000xg for 15 min) was additionally filtered through Miracloth and transferred onto a sucrose cushion solution (0.4 M Tris pH 7.4, 0.2 M KCl, 0.005 M EGTA, 0.035 M MgCl_2_, 1.75 M sucrose, 5 mM DTT, 100 μg/mL cyclohexamide, 100 μg/mL chloramphenicol) and centrifuged at 4 °C, 170,000xg for 3 h. The ribosome-containing pellet was gently resuspended in ice-cold resuspension buffer (0.2 M Tris pH 7.4, 0.2 M KCl, 0.025 M EGTA, 0.035 M MgCl_2_, 5 mM DTT, 100 μg/mL cyclohexamide, 100 μg/mL chloramphenicol) and applied onto 15 – 60 % sucrose gradient (0.04 M Tris pH 7.4, 0.02 M KCl, 0.01 M MgCl_2_, 100 μg/mL cyclohexemide, 100 μg/mL chloramphenicol) and centrifuged at 4 °C, 237,000xg for 1.5 h.

### Preparation of RPF and total mRNA libraries

Purified polysomes were digested with RNAse I (1.5U/1OD/1 μl) at 22 °C for 10 min, loaded directly onto 15–60 % sucrose gradient and centrifuged at 4 °C, 237,000xg for 1.5 h. The amount of loaded sample was normalized according to the input material, hence the variations in the samples mirror the different RNA content of each sample. The monosome fraction was concentrated with an Amicon-Ultra4 Centrifugal Unit (MWL 100 kDa) and RPFs were released by adding the release buffer (20 mM HEPES-KOH pH 7.4, 100 mM KCl, 1 mM EDTA, 2 mM DTT, 2 μl/ml Ribolock) and incubated for 10 min on ice and centrifuged at 4 °C, 1900xg for 30 min. RNA was extracted using the hot acid phenol method and depleted of rRNA using RiboMinus Plant Kit (Ambion). Samples were normalized on the input material.

Total RNA was extracted using TRIzol reagent and spiked with ERCC RNA Spike-In Mix (Ambion). rRNA was depleted using RiboMinus Plant Kit (Ambion), and randomly fragmented by alkaline lysis in alkaline fragmentation solution (2 mM EDTA, 12 mM Na_2_CO_3_, 87 mM NaHCO_3_) at 95 °C for 40 min. The randomly fragmented RNA was recovered by precipitation in the presence of glycogen.

The sequencing libraries were prepared according to [16]. Briefly, both randomly fragmented total RNA and RPF were loaded onto a 15 % TBE-polyacrylamide gel (containing 8 M urea). RNA fragments with a size of 25–35 nucleotides, which size corresponds to a nucleotide sequence covered by the ribosomes [16], were cut out of the gel and isolated by centrifugation at 17,000xg for 5 min to crush the gel, eluted by incubating with 3 M Na acetate buffer (pH 5.5) containing glycogen and RiboLock (Thermo Fischer Scientific) for 4 h at 4 °C and purified by precipitation with isopropanol. To those fragments 5′ and 3′ adaptors were ligated and subjected to deep sequencing on the Illumina Hiseq2000 platform.

### Mapping of the sequences and reads distribution analysis

The sequencing data was mapped against the TAIR 10 annotated *A. thaliana* genome (downloaded from ENSEMBL, version 21) using Bowtie 1.0.0. Perfectly mapped reads (i.e., without any mismatch) to an rRNA reference were discarded after the first mapping round. The remaining reads were the mapped to the genome, the parameters were adjusted according to the properties of very short reads (-v 2 -m 1 --strata --best -y) and only uniquely mapped reads were kept for further analysis. The number of raw reads unambiguously aligned to ORFs in both RNA-Seq and ribosome profiling data sets from two biological replicates were used to generate gene read counts, which were normalized as reads per million of the total mapped reads (rpm) or reads per kilobase per million of the total mapped reads (rpkm) [17].

### Differential expression and enrichment analysis

Mapped read counts were applied to protein coding transcripts using the longest annotated transcript for each AGI identifier. The detection limit (2 rpkm) in each experiment was determined from the linear range of detection of the Spike-In Mix and the selected reliably expressed transcripts were subjected to differential expression analysis by means of DESeq (version 1.16.0; [54]) using a false discovery rate of 0.1. Enrichment analyses were performed using DAVID [55, 56].

### Secondary structure analysis, motif search and analysis for putative G-quadruplex structures

Secondary structure of the 5′ UTRs and coding mRNAs was computed with RNAfold program (2.1.7; default parameters) from the ViennaRNA Package 2.0 [57] using a sliding window of 39 nt and assigning the minimal free energy to the middle nucleotide [35]. Average profiles for different gene groups were generated by taking the mean of their per-base folding energy contributions.

To identify conserved motifs in the sequences, we used MEME version 4.10.0_4 (available online at http://meme-suite.org/tools/meme). The parameters were set as motif minimal width of 6, motif maximal width of 20, maximal numbers of motifs 10 and ‘zero or one per sequence’.

The position of potential G-quadruplexes were predicted using custom R-scripts which analyzed the sequences for presence of G2-, G3- and G4-quadruplexes sequences matching the pattern G_2_N_1-7_G_2_N_1-7_G_2_N_1-7_G_2_, G_3_N_1-7_G_3_N_1-7_G_3_N_1-7_G_3_ and G_4_N_1-7_G_4_N_1-7_G_4_N_1-7_G_4_, respectively.

### Analysis of the protein production in chloroplasts

Protein production was determined using only the normalized RPF reads in the CDS normalized to the mRNA reads as described by Li et al. [30]. The protein abundance data we obtained were compared with the protein production in chloroplasts determined with mass spectrometry by Baginsky and colleagues [31]. The data from the two measurements [31] were averaged and only proteins present in both were used.

## Additional files

Additional file 1: Correlation of the sequencing data. (a, b) Correlation of the mRNA (a) and RPF (b) read counts. Only genes over the detection limit, determined by the spike-ins, in both Ribo-Seq and RNA-Seq are included. r, Pearson correlation coefficients. (c, d) Correlation of the normalized RPF and randomly fragmented mRNA counts for each gene from control plants (c) or plants subjected to heat stress (d). r, Pearson correlation coefficients. (e) Polysomal profiles of plants grown at permissive ambient temperature (blue) and upon exposure to heat stress for 3 h (red). Total RNA loaded on the gradients was normalized according to the mass of the material used for polysome purification. The profiles changed marginally for stress-exposed plants: the polysome fraction slightly decreased under stress with no increase of the monosomes suggesting fully functional translation. r, Person correlation coefficient. (f) Different groups of RNAs were detected in the samples. As expected, in the RPF samples around 90 % of the reads were mapped to protein-coding genes. Protein-coding genes were also feature to which the most RNA reads from the Ribo-Seq were mapped. In both types of samples, tRNA was the second most abundant group, reaching roughly 10 % in the RPF samples and 40 % in the RNA samples. (PDF 1414 kb)
Additional file 2: Comparison of transcriptional and translational features of all protein-coding genes between control plants and those exposed to thermal stress. (a, b) Correlation of the normalized RPF (a) and randomly fragmented mRNA reads (b) for each gene from control plants or plants subjected to heat stress. (c, d) Symmetric distribution of the mRNA (black) and RPF (red) reads between the first and second halves of the CDS of each transcript for control (c) and heat stress (d). r, Pearson correlation coefficients. (PDF 1414 kb)
Additional file 3: GO term analysis of the genes translationally and transcriptionally altered upon heat exposure for the genes groups for which changes with DESeq were detected (Fig. 1b). Cluster enrichment score represents the overall enrichment for a group in the input lists of terms and thus is informative of the most frequent GO terms. The horizontal lines mark the clusters. (PDF 1414 kb)
Additional file 4: The mRNA levels of marker stress-related genes are upregulated under thermal stress. (a) Quantification of mRNA expression of marker genes upregulated by heat by means of qRT-PCR (white bars; mean ± SEM, *n* = 3). For comparison the mRNA expression in our RNA-Seq data set (gray bars) and microarray data (dark gray bars; [7]) is given. In all three representations, the mRNA expression values are presented as a fold change (log_2_) compared to the control plants. In qRT-PCR data the mRNA expression values were normalized to the expression of housekeeping gene UBQ10. (b) mRNA (gray) and RPF (red) coverage profiles of the stress marker genes under control and thermal stress conditions. The schematic of each gene is included; exons are designated in black. (PDF 1414 kb)
Additional file 5: The expression level of genes with G2 and G3 quadruplexes in the CDS do not change upon stress exposure. (a, b) The expression level in the vicinity of the putative G2 (a) or G3 (b) quadruplexes in the CDS is not changed between heat stress-exposed (red) and the control plants (blue). RPF coverage (rpm) was normalized to the mRNA reads at each position and each gene in the set is equally weighted. The first nucleotide of the G2 quadruplexes is at position 200. *p*-values on the top of the plots were calculated with Wilcoxon signed rank sum test. (PDF 1414 kb)
Additional file 6: Heat stress-induced changes in the production of protein subunits of the plastid protein complexes. (a, b) Expression level and stoichiometry of the subunits in various chloroplast-encoded protein complexes in control plants (a) and in plants exposed to heat stress (b). Genes encoded within one operon are shown in the same color. (PDF 1414 kb)

## Abbreviations

bp: Basepairs
CDS: Coding sequence
nt: Nucleotide
ORF: Open-reading frame
RPF: Ribosome-protected fragments
