# Supplementary material for: Insights into the adaptive response of Arabidopsis thaliana to prolonged thermal stress by ribosomal profiling and RNA-Seq
Source: BMC Plant Biol. 2016 Oct 10;16:221. doi: 10.1186/s12870-016-0915-0 (PMC5057212; doi:10.1186/s12870-016-0915-0)
Supplement: Additional file 1: — Correlation of the sequencing data. (a, b) Correlation of the mRNA (a) and RPF (b) read counts. Only genes over the detection limit, determined by the spike-ins, in both Ribo-Seq and RNA-Seq are included. r, Pearson correlation coefficients. (c, d) Correlation of the normalized RPF and randomly fragmented mRNA counts for each gene from control plants (c) or plants subjected to heat stress (d). r, Pearson correlation coefficients. (e) Polysomal profiles of plants grown at permissive ambient temperature (blue) and upon exposure to heat stress for 3 h (red). Total RNA loaded on the gradients was normalized according to the mass of the material used for polysome purification. The profiles changed marginally for stress-exposed plants: the polysome fraction slightly decreased under stress with no increase of the monosomes suggesting fully functional translation. r, Person correlation coefficient. (f) Different groups of RNAs were detected in the samples. As expected, in the RPF samples around 90 % of the reads were mapped to protein-coding genes. Protein-coding genes were also feature to which the most RNA reads from the Ribo-Seq were mapped. In both types of samples, tRNA was the second most abundant group, reaching roughly 10 % in the RPF samples and 40 % in the RNA samples. (PDF 1414 kb) [file 12870_2016_915_MOESM1_ESM.pdf]

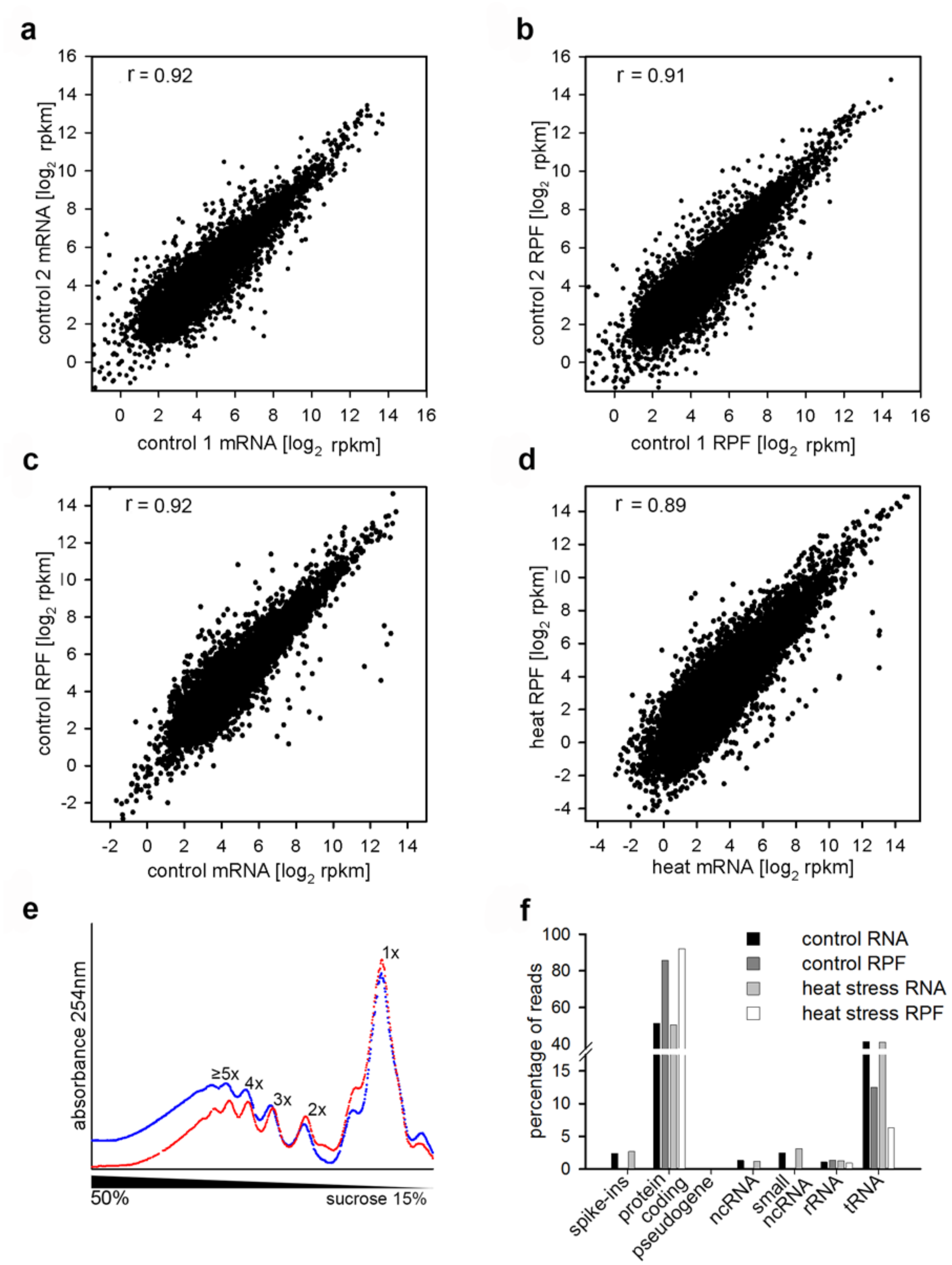

Additional File 1. Correlation of the sequencing data. (**a, b**) Correlation of the mRNA (a) and RPF (b) read counts. Only genes over the detection limit, determined by the spike-ins, in both Ribo-Seq and RNA-Seq are included. r, Pearson correlation coefficients. (**c, d**) Correlation of

the normalized RPF and randomly fragmented mRNA counts for each gene from control plants (**c**) or plants subjected to heat stress (**d**). r, Pearson correlation coefficients. (**e**) Polysomal profiles of plants grown at permissive ambient temperature (blue) and upon exposure to heat stress for 3h (red). Total RNA loaded on the gradients was normalized according to the mass of the material used for polysome purification. The profiles changed marginally for stress-exposed plants: the polysome fraction slightly decreased under stress with no increase of the monosomes suggesting fully functional translation. r, Person correlation coefficient. (**f**) Different groups of RNAs were detected in the samples. As expected, in the RPF samples around 90% of the reads were mapped to protein-coding genes. Protein-coding genes were also feature to which the most RNA reads from the Ribo-Seq were mapped. In both types of samples, tRNA was the second most abundant group, reaching roughly 10% in the RPF samples and 40% in the RNA samples.
