# Supplementary material for: Insights into the adaptive response of Arabidopsis thaliana to prolonged thermal stress by ribosomal profiling and RNA-Seq
Source: BMC Plant Biol. 2016 Oct 10;16:221. doi: 10.1186/s12870-016-0915-0 (PMC5057212; doi:10.1186/s12870-016-0915-0)
Supplement: Additional file 2: — Comparison of transcriptional and translational features of all protein-coding genes between control plants and those exposed to thermal stress. (a, b) Correlation of the normalized RPF (a) and randomly fragmented mRNA reads (b) for each gene from control plants or plants subjected to heat stress. (c, d) Symmetric distribution of the mRNA (black) and RPF (red) reads between the first and second halves of the CDS of each transcript for control (c) and heat stress (d). r, Pearson correlation coefficients. (PDF 1414 kb) [file 12870_2016_915_MOESM2_ESM.pdf]

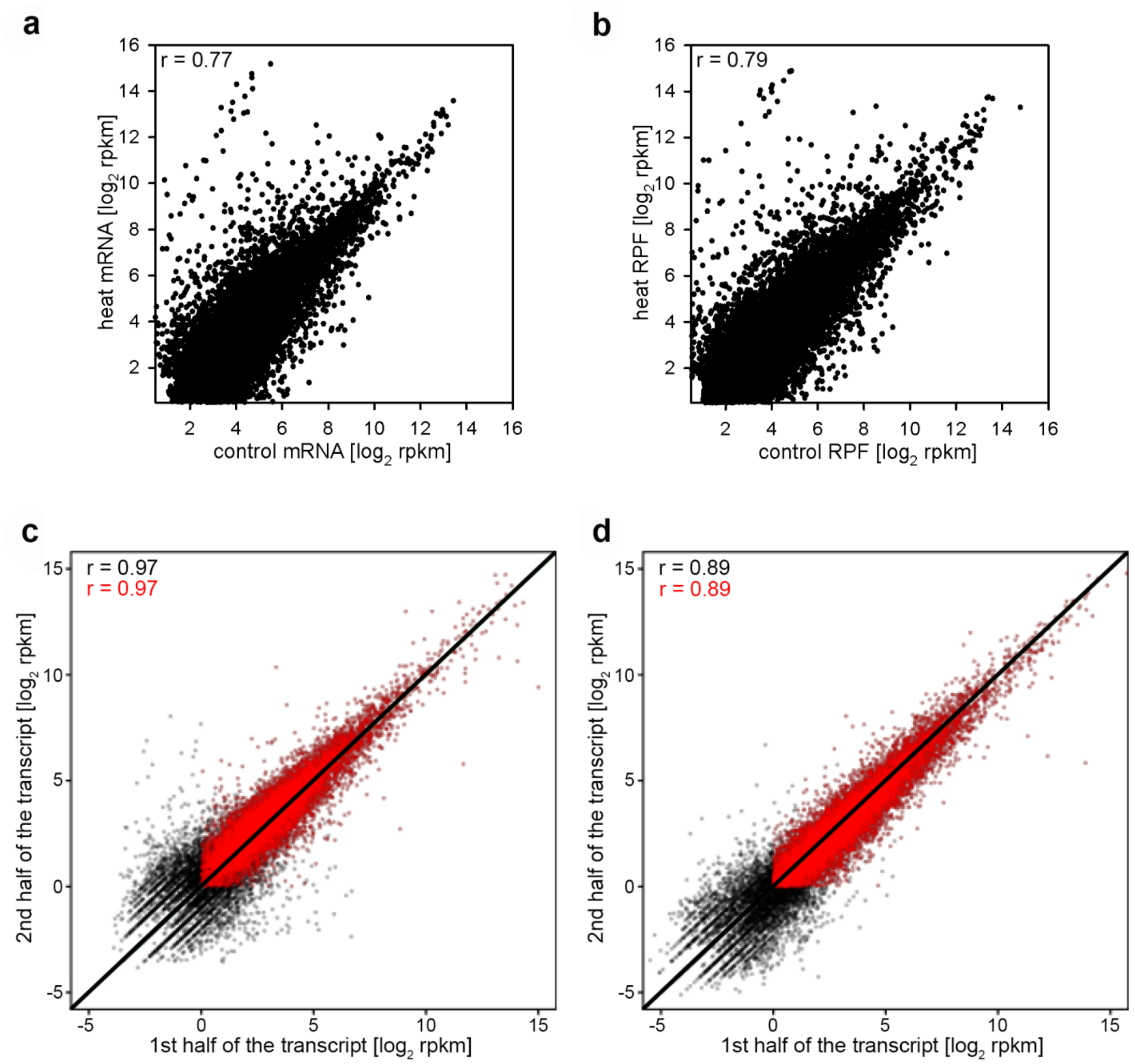

Additional File 2. Comparison of transcriptional and translational features of all protein-coding genes between control plants and those exposed to thermal stress. (**a, b**) Correlation of the normalized RPF (a) and randomly fragmented mRNA reads (b) for each gene from control plants or plants subjected to heat stress. (**c, d**) Symmetric distribution of the mRNA (black) and RPF (red) reads between the first and second halves of the CDS of each transcript for control (c) and heat stress (d). r, Pearson correlation coefficients.
