# Supplementary material for: Insights into the adaptive response of Arabidopsis thaliana to prolonged thermal stress by ribosomal profiling and RNA-Seq
Source: BMC Plant Biol. 2016 Oct 10;16:221. doi: 10.1186/s12870-016-0915-0 (PMC5057212; doi:10.1186/s12870-016-0915-0)
Supplement: Additional file 3: — GO term analysis of the genes translationally and transcriptionally altered upon heat exposure for the genes groups for which changes with DESeq were detected (Fig. 1b). Cluster enrichment score represents the overall enrichment for a group in the input lists of terms and thus is informative of the most frequent GO terms. The horizontal lines mark the clusters. (PDF 1414 kb) [file 12870_2016_915_MOESM3_ESM.pdf]

GO-term both down	Count	P-value	Cluster enrichment score
nucleosome	7	1.05E-08	4.681
protein-DNA complex	7	2.00E-08	
nucleosome assembly	7	2.37E-08	
nucleosome organization	7	2.37E-08	
chromatin assembly	7	2.82E-08	
protein-DNA complex assembly	7	3.07E-08	
DNA packaging	7	4.61E-08	
chromatin	7	9.77E-08	
chromatin assembly or disassembly	7	1.44E-07	
chromosomal part	7	2.37E-06	
chromosome	7	1.35E-05	
cellular macromolecular complex	7	1.59E-05	
assembly	7	2.12E-05	
chromatin organization	7	2.54E-05	
cellular macromolecular complex subunit			
organization	7	5.32E-05	
chromosome organization	7	6.85E-05	
macromolecular complex assembly	7	9.97E-05	
macromolecular complex subunit			
organization			
cellular amino acid biosynthetic process	4	1.25E-02	1.565
amine biosynthetic process	4	1.65E-02	
organic acid biosynthetic process	5	2.20E-02	
carboxylic acid biosynthetic process	5	2.20E-02	
cellular carbohydrate biosynthetic	5	1.50E-03	1.535
process	5	4.37E-03	
carbohydrate biosynthetic process			

GO-term mRNA down RPF 0	Count	P-value	Cluster enrichment score
cell wall	7	3.45E-03	2.089
extemRNAl encapsulating structure	7	3.71E-03	
cell wall modification	4	4.22E-03	
extracellular region	9	8.53E-03	
plant-type cell wall	4	2.85E-02	
cell wall organization	4	2.92E-02	
extemRNAl encapsulating structure organization	4	3.37E-02	
defense response	7	3.57E-02	1.170
response to light stimulus	5	2.55E-02	1.108
response to radiation	5	2.85E-02	
response to gibberellin stimulus	3	3.37E-02	

GO-term mRNA 0 RPF up	Count	P-value	Cluster enrichment score
response to abiotic stimulus	28	1.03E-03	2.237
response to temperature stimulus	12	3.66E-03	
response to osmotic stress	12	8.77E-03	
response to salt stress	10	3.41E-02	
cofactor biosynthetic process	26	3.07E-03	1.895
iron-sulfur cluster assembly	22	2.59E-02	
metallo-sulfur cluster assembly	19	2.59E-02	

GO-term both up	Count	P-value	Cluster enrichment score
response to heat	65	5.14E-74	50.425
response to temperature stimulus	75	6.80E-53	
response to abiotic stimulus	100	4.89E-34	
protein folding	52	1.62E-35	24.236
unfolded protein binding	23	3.43E-16	
response to high light intensity	21	2.40E-22	13.774
response to inorganic substance	52	1.49E-19	
response to light intensity	22	1.69E-18	
response to oxidative stress	32	1.05E-13	
response to reactive oxygen species	22	2.12E-12	
response to hydrogen peroxide	20	5.76E-12	
response to light stimulus	33	6.06E-09	
response to radiation	33	1.36E-08	

GO-term mRNA up RPF 0	Count	P-value	Cluster enrichment score
intracellular organelle lumen	8	1.15E-03	2.096
organelle lumen	8	1.15E-03	
membrane-enclosed lumen	8	1.24E-03	
nuclear lumen	6	6.41E-03	
non-membrane-bounded organelle	8	2.29E-02	
intracellular non-membrane-bounded organelle	8	2.29E-02	
ATPase activity	6	2.72E-02	1.214
ribonucleotide binding	16	4.44E-02	
purine ribonucleotide binding	16	4.44E-02	
nucleotide binding	19	4.49E-02	
ATPase activity	6	2.72E-02	1.181
ATPase activity, coupled	5	3.78E-02	

GO-term mRNA 0 RPF down	Count	P-value	Cluster enrichment score
microtubule motor activity	14	3.76E-15	9.970
microtubule-based movement	14	6.01E-15	
microtubule-based process	16	6.29E-15	
microtubule	17	8.54E-15	
motor activity	15	2.05E-14	
microtubule cytoskeleton	18	6.37E-14	
cytoskeletal part	19	9.08E-14	
cytoskeleton	19	5.65E-12	
microtubule associated complex	10	1.09E-09	
ATP binding	37	2.95E-05	
adenyl ribonucleotide binding	37	3.82E-05	
purine ribonucleotide binding	37	3.18E-04	
ribonucleotide binding	37	3.18E-04	
non-membrane-bounded organelle	23	5.17E-04	
intracellular non-membrane-bounded organelle	23	5.17E-04	
calmodulin binding	10	9.45E-06	3.560
cell cycle process	8	4.58E-05	2.750
cell cycle	9	2.68E-04	
M phase	5	2.45E-03	
cell cycle phase	5	4.08E-03	
cell division	6	7.42E-03	
cytokinesis	3	3.46E-02	

Additional File 3. GO term analysis of the genes translationally and transcriptionally altered upon heat exposure for the genes groups for which changes with DESeq were detected (Fig. 1b). Cluster enrichment score represents the overall enrichment for a group in the input lists of terms and thus is informative of the most frequent GO terms. The horizontal lines mark the clusters.
