# Supplementary material for: Insights into the adaptive response of Arabidopsis thaliana to prolonged thermal stress by ribosomal profiling and RNA-Seq
Source: BMC Plant Biol. 2016 Oct 10;16:221. doi: 10.1186/s12870-016-0915-0 (PMC5057212; doi:10.1186/s12870-016-0915-0)
Supplement: Additional file 4: — The mRNA levels of marker stress-related genes are upregulated under thermal stress. (a) Quantification of mRNA expression of marker genes upregulated by heat by means of qRT-PCR (white bars; mean ± SEM, n = 3). For comparison the mRNA expression in our RNA-Seq data set (gray bars) and microarray data (dark gray bars; [7]) is given. In all three representations, the mRNA expression values are presented as a fold change (log2) compared to the control plants. In qRT-PCR data the mRNA expression values were normalized to the expression of housekeeping gene UBQ10. (b) mRNA (gray) and RPF (red) coverage profiles of the stress marker genes under control and thermal stress conditions. The schematic of each gene is included; exons are designated in black. (PDF 1414 kb) [file 12870_2016_915_MOESM4_ESM.pdf]

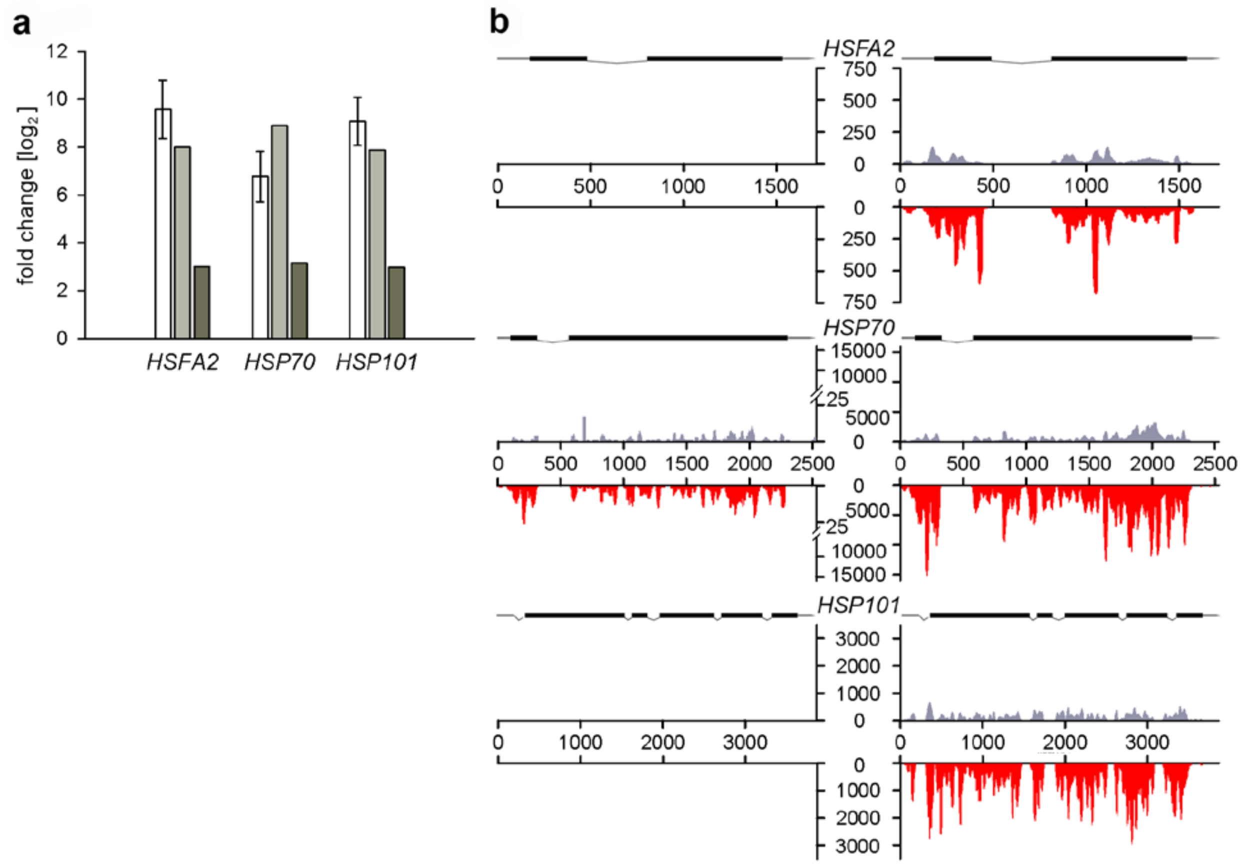

Additional File 4. The mRNA levels of marker stress-related genes are upregulated under thermal stress. (**a**) Quantification of mRNA expression of marker genes upregulated by heat by means of qRT-PCR (white bars; mean ± SEM, n = 3). For comparison the mRNA expression in our RNA-Seq data set (gray bars) and microarray data (dark gray bars; [7]) is given. In all three representations, the mRNA expression values are presented as a fold change (log_2) compared to the control plants. In qRT-PCR data the mRNA expression values were normalized to the expression of housekeeping gene UBQ10. (**b**) mRNA (gray) and RPF (red) coverage profiles of the stress marker genes under control and thermal stress conditions. The schematic of each gene is included; exons are designated in black.
