# Supplementary material for: Insights into the adaptive response of Arabidopsis thaliana to prolonged thermal stress by ribosomal profiling and RNA-Seq
Source: BMC Plant Biol. 2016 Oct 10;16:221. doi: 10.1186/s12870-016-0915-0 (PMC5057212; doi:10.1186/s12870-016-0915-0)
Supplement: Additional file 5: — The expression level of genes with G2 and G3 quadruplexes in the CDS do not change upon stress exposure. (a, b) The expression level in the vicinity of the putative G2 (a) or G3 (b) quadruplexes in the CDS is not changed between heat stress-exposed (red) and the control plants (blue). RPF coverage (rpm) was normalized to the mRNA reads at each position and each gene in the set is equally weighted. The first nucleotide of the G2 quadruplexes is at position 200. p-values on the top of the plots were calculated with Wilcoxon signed rank sum test. (PDF 1414 kb) [file 12870_2016_915_MOESM5_ESM.pdf]

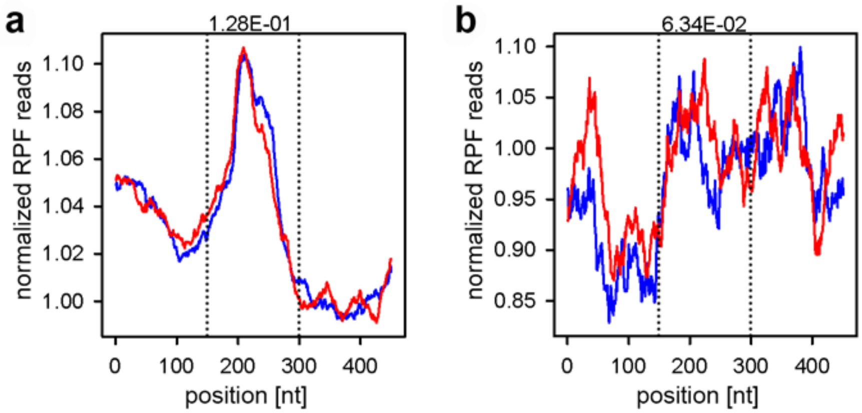


Additional File 5**.** The expression level of genes with G2 and G3 quadruplexes in the CDS do not change upon stress exposure. (**a, b**) The expression level in the vicinity of the putative G2 (a) or G3 (b) quadruplexes in the CDS is not changed between heat stress-exposed (red) and the control plants (blue). RPF coverage (rpm) was normalized to the mRNA reads at each position and each gene in the set is equally weighted. The first nucleotide of the G2 quadruplexes is at position 200. *p*-values on the top of the plots were calculated with Wilcoxon signed rank sum test.
