# Supplementary material for: Insights into the adaptive response of Arabidopsis thaliana to prolonged thermal stress by ribosomal profiling and RNA-Seq
Source: BMC Plant Biol. 2016 Oct 10;16:221. doi: 10.1186/s12870-016-0915-0 (PMC5057212; doi:10.1186/s12870-016-0915-0)
Supplement: Additional file 6: — Heat stress-induced changes in the production of protein subunits of the plastid protein complexes. (a, b) Expression level and stoichiometry of the subunits in various chloroplast-encoded protein complexes in control plants (a) and in plants exposed to heat stress (b). Genes encoded within one operon are shown in the same color. (PDF 1414 kb) [file 12870_2016_915_MOESM6_ESM.pdf]

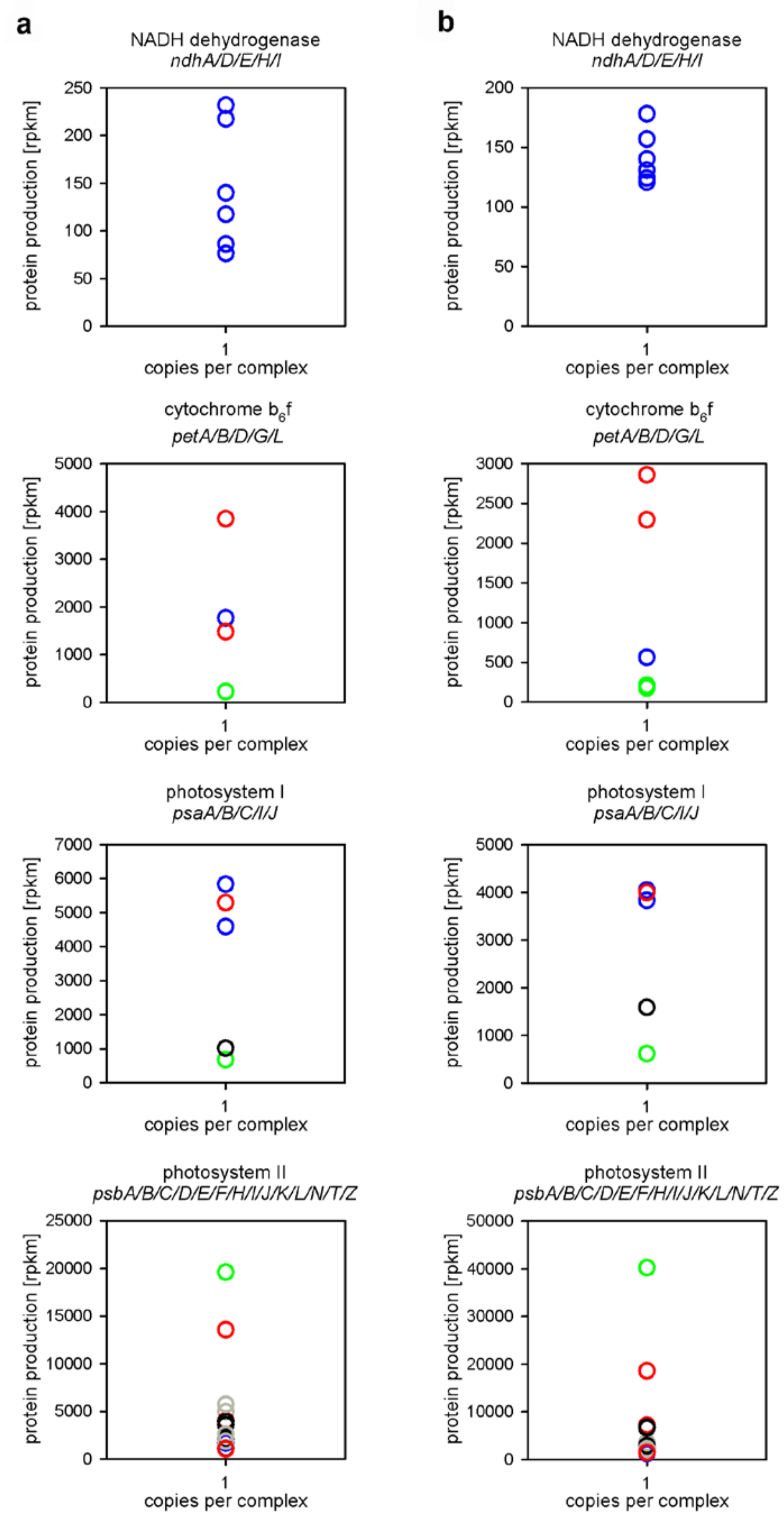
a
b
NADH dehydrogenase
ndhA/D/E/H/I
cytochrome b6f
petA/B/D/G/L
photosystem I
psaA/B/C/I/J
photosystem II
psbA/B/C/D/E/F/H/I/J/K/L/N/T/Z
protein production [rpkm]
copies per complex
1

Additional File 6. Heat stress-induced changes in the production of protein subunits of the plastid protein complexes. (**a, b**) Expression level and stoichiometry of the subunits in various chloroplast-encoded protein complexes in control plants (a) and in plants exposed to heat stress (b). Genes encoded within one operon are shown in the same color.
